# Compositional and functional changes in the salivary microbiota related to oral leukoplakia and oral squamous cell carcinoma: a case control study

**DOI:** 10.1186/s12903-023-03760-y

**Published:** 2023-12-19

**Authors:** Qingying Lan, Chang Zhang, Hong Hua, Xiaosheng Hu

**Affiliations:** Department of Oral Medicine, National Center of Stomatology & National Clinical Research Center for Oral Diseases & National Engineering Laboratory for Digital and Material Technology of Stomatology & Beijing Key Laboratory of Digital Stomatology & Research Center of Engineering and Technology for Computerized Dentistry Ministry of Health & NMPA Key Laboratory for Dental Materials, Peking University School and Hospital of Stomatology, 22 South Zhongguancun Avenue, Haidian District, Beijing, 100081 China

**Keywords:** Metagenomic sequencing, Oral squamous cell carcinoma, Oral leukoplakia, Oral microbiome, Saliva, Gemella

## Abstract

**Background:**

Oral squamous cell carcinoma (OSCC) is one of the most common malignant tumours with increasing incidence, and oral leukoplakia (OLK) has a strong tendency to undergo malignant transformation. The oral microbiota may influence oral cancer progression, but the salivary bacterial composition and functional changes in OSCC and OLK have not been comprehensively elucidated. Therefore, we compared salivary bacteria in OLK and OSCC patients with healthy controls (HC).

**Methods:**

Metagenomic sequencing was used to compare the bacterial composition and functional changes of 18 OSCC patients, 21 OLK patients and 21 HC. Spearman correlation was used to identify possible associations between functions and bacteria.

**Results:**

*Gemella* was the most differentially enriched genus in OSCC. At the species level, *Streptococcus* sp. NPS 308, *Streptococcus agalactiae*, *Gemella haemolysans* and *Gemella morbillorum* were slightly increased in OLK and OSCC. Kyoto Encyclopedia of Genes and Genomes (KEGG) results showed that OSCC was mainly associated with metabolism functions, including lipid metabolism, carbohydrate metabolism and glycan biosynthesis and metabolism. The synthesis and degradation of ketone bodies, cysteine and methionine metabolism and glycerolipid metabolism differed significantly among the three groups, and were highest in OSCC and lowest in HC. And *G. haemolysans* was significantly associated with these selected metabolic pathways.

**Conclusions:**

Metagenomic analysis revealed significant differences in the salivary microbiota among OSCC, OLK and HC. Thus, salivary microbiota composition and functional changes may be associated with OSCC progression. Metabolism of nonessential amino acids such as cysteine and methionine in bacteria may play an important role in oral oncogenesis, and more studies of the mechanism between metabolisms of bacteria and oral oncogenesis are needed in the future.

**Supplementary Information:**

The online version contains supplementary material available at 10.1186/s12903-023-03760-y.

## Introduction

Oral cancer is increasing in incidence, and ~ 377,713 new cases were diagnosed in 2020, which > 90% was oral squamous cell carcinoma (OSCC) worldwide [1, 2]. Furthermore, OSCC has a 5-year survival rate of ~ 50% worldwide [3]. Oral leukoplakia (OLK) is one of the most common types of oral potentially malignant disorders (OPMDs), with a malignant transformation (MT) rate of 9.8% (95% CI: 7.9−11.7) [4]. However, sensitive non-invasive examinations to screen MT of OLK were still lacking [5].

Bacterial infections contribute to carcinogenesis. For example, *Helicobacter pylori* promotes the development of gastric cancer through epithelial injury and inflammation [6]. Possible associations between cervical cancer and microbiomes have been reported for *Peptostreptococcus* and *Enterococcus* [7]. Regarding oral microbiomes, *Porphyromonas gingivalis* [8] and *Fusobacterium nucleatum* [9] were found to promote the development of colorectal carcinoma. Microbe-associated molecular patterns, metabolites derived by microbe may interact with host cell surface molecules and induce immune responses for virus infection [10].

Genetic changes, smoking, betel nut chewing and heavy alcohol consumption are considered the main risk factors of OSCC and OLK, but ~ 15% of cases cannot be explained by these factors [11–13]. Researchers are increasing discovering that bacterial, viral and fungal infections may be potential risk factors of oral tumours as well as OLK [14, 15]. Such potential associations have been explored, but mainly based analysis of the 16S ribosomal RNA (16S rRNA). Saliva examination has the advantages of non-invasion and easy nature of collection. Our previous study reported higher diversity in OSCC than OLK and healthy control (HC) groups, and bacterial metabolism may related to those changes [16]. Another study of saliva samples found that Bacteriodetes members were more abundant in OLK [17]. Some researchers found that Firmicutes (especially *Streptococcus*) [18] and Actinobacteria (especially *Rothia*) [18] were significantly decreased in OSCC lesions compared with normal tissues from the same patients, while *Porphyromonas* (in whole mouth fluid) [17] and Fusobacteria (in saliva) [19] were significantly more abundant in OSCC. These studies mainly explored differences at phylum and genus levels [20], but a consensus was not reached, and potential functional differences in microbiomes that may be associated with OSCC and OLK remain largely unexplored. Metagenomic analyses can provide functional information that reveals the underlying mechanisms linking microbiome interactions and diseases [21]. Although some metagenomic analyses have been conducted on OSCC and OLK separately, simultaneous investigations are lacking.

In this study, we characterized the salivary microbiome in OLK, OSCC and HC groups using microbial metagenomic analysis. We investigated bacterial shifts and their associations with pathological changes. Additionally, we explored changes in microbiome function across the three groups to uncover potential connections among diseases, microbiomes, and functional alterations. The results can provide a new perspective for exploring the pathogenesis and diagnosis of OLK and OSCC.

## Materials and methods

### Study design

This study was approved by the Ethics Committee of the Peking University School of Stomatology (registration number PKUSSIRB-201733020). All participants signed informed consent forms before participation. A total of 18 OSCC, 21 OLK and 21 HC individuals were enrolled in the study, and 60 saliva samples were collected from the Department of Oral Medicine, Peking University School and Hospital of Stomatology, Beijing, China, from September 2019 to March 2021. OSCC patients were diagnosed and confirmed clinically and pathologically (International Classification of Diseases, 10th revision [ICD-10], codes C02–C06). OLK patients were diagnosed and confirmed by oral medicine professionals and oral pathologists using 2018 WHO criteria [22]. Besides, architectural changes (tissue changes) and cytological changes (individual cell changes/cytological atypia) were defined as epithelial dysplasia (ED) (WHO classification of tumours of the head and neck, 4th revision, 2017) [22, 23]. An increase of cell number and cellular atypia were divided into hyperplasia/keratosis (HK) (WHO classification of tumours of the head and neck, 4th revision, 2017) [22, 23]. All pathological diagnoses were confirmed by two pathologists and all clinical diagnoses were confirmed by two oral medicine specialists. Finally, 9 patients of OLK were included in ED subgroup, and 12 in HK subgroup. Volunteers without tumours, oral mucosal diseases based on physical examination were enrolled as the HC group. All saliva samples were collected and subjected to metagenomic sequencing analysis. Basic information on patients including age and sex was also recorded.

### Inclusion and exclusion criteria

All participants were over 18 years old of age and volunteered to participate and cooperate with the investigators. Included subjects were allocated to OLK, OSCC or HC groups. The patient group inclusion criteria were as follows: (1) pathological diagnosis of OSCC or OLK; (2) absence of other mucosal lesions in the oral cavity; (3) absence of suspicion that oral lesions may be related to any drug or oral restoration. The exclusion criteria were as follows: (1) antibiotics taken within 1 month; (2) treatments with radiotherapy and chemotherapy; (3) other malignant tumours or a history of malignant tumours; (4) pregnant or lactating women or minors (< 18 years of age); (5) unable to cooperate with investigators for any reasons.

### Sample collection and processing, saliva DNA extraction, library construction, metagenomic sequencing, taxonomic profiling and functional annotation

A standard technique of saliva collection was carried out as described by Navazesh (1993) [24]. All subjects were asked to refrain from smoking, drinking or eating for at least 1 h before sample collection. And all saliva samples were collected before surgery or biopsy. A 2−3 ml volume of non-stimulated saliva was collected into a 50-ml sterile falcon tube from each subject. Saliva samples were centrifuged at 10,000 g for 15 min and pellets were stored at -80℃ for further analysis. The saliva samples were sent to Wekemo Tech Group Co. Ltd. (Shenzhen, China) for metagenomic sequencing and data analysis. And all the sequencing was run in one batch. Total DNA was extracted from samples using the cetyltrimethylammonium bromide (CTAB) extraction method. DNA purity and integrity were determined using a Qubit Fluorometric Quantification (Life Technologies, Carlsbad, CA, USA) and by agarose gel electrophoresis. A Next Ultra DNA Library Prep Kit (NEB, Ipswich, Suffolk, UK) was used to construct DNA libraries according to the manufacturer’s protocol. A NovaSeq platform (Illumina, San Diego, CA, USA) was employed to carry out two-terminal sequencing of samples following a 2 × 150 bp paired-end read protocol. Trimmomatic was used for trimming and quality control [25]. Bowtie 2 was used to remove the host sequence (parameter: very sensitive) [26]. Through the above procedure, clean data were obtained for subsequent analysis. Fast QC was used to check the rationality and the effect of quality control before and after quality control. Kraken2 [27] (parameter: confidence 0.2) was used to annotate and classify all valid sequences of samples. The number of raw reads was 19,630,322 to 33,082,182 (mean, 22,992,086 ± 3,361,764) before removing the host sequence. And after removing the host sequence, the number of clean reads was 223,339 to 23,976,716 (mean, 6,959,630 ± 6,736,291). After using Kraken, Bracken [28] (parameter: default) was used to classify the results obtained from Kraken2, and Bayesian was used to estimate the abundance of each sample at different phylogenetic levels (phylum, class, order, family, genus and specie) [29, 30].

HUMAnN2 software (published in Nature methods in 2018) was used to compare the obtained sequences with those in the protein database (UniRef90; based on DIAMOND, version 0.7.10.59) [31]. According to the corresponding relationship between the UniRef90 ID and the Kyoto Encyclopedia of Genes and Genomes (KEGG) database ID (mainly from LinkDB), the prevalence of functions was determined. This report conforms to the Strengthening the Reporting of Observational Studies in Epidemiology (STROBE) guidelines [32].

### Statistical analysis

Bioinformatic analysis was performed using Wekemo Bioincloud (https://www.bioincloud.tech). Statistical analyses were performed using SPSS software (version 26.0; IBM, Armonk, NY, USA) and OriginLab (version 9.8; OriginLab Corporation, Northampton, Massachusetts, USA). One-way analysis of variance (ANOVA) and Kruskal-Wallis H tests were used to compare continuous variables in multiple comparisons. Differences between two groups were analysed by Dunn tests. Alpha-diversity of Shannon, Ace and Chao1 indices was calculated using Wekemo Bioincloud. Beta-diversity was assessed using principal coordinate analysis (PCoA; Bray-Curtis algorithm). Permutational multivariate analysis of variance (PERMANOVA) was applied to quantify the proportion of variation in taxonomy and pathway profiles explained by disease status and potential cofounding variables. Linear discriminant analysis (LDA) effect size (LEfSe) analysis was employed for community functional and compositional structure comparisons, and the logarithmic LDA score cut-off was set to 2.0. Correlation tests were performed using Spearman rank correlation analysis. In all tests, *p* < 0.05 was considered statistically significant.

## Results

### Characteristics of the primary cohort

Final analysis and metagenomic sequencing of samples were carried out for 60 subjects spanning OSCC, OLK and HC groups (n = 18, 21 and 21, respectively). The average age of the HC group was 48.81 ± 12.38 (7 males and 14 females), compared with 59.24 ± 12.30 for OLK (4 males and 17 females) and 54.11 ± 15.43 for OSCC (8 males and 10 females). The habits of HC, OLK and OSCC were collected, including smoking (n = 2, 3, 5) and alcohol (n = 2, 2, 5). And none of the participants had betel chewing habits. The clinical characteristics of the study participants are listed in Additional file 1.

### Taxonomic characteristics of OSCC, OLK and HC groups

#### Abundance summary

**Fig. 1:**
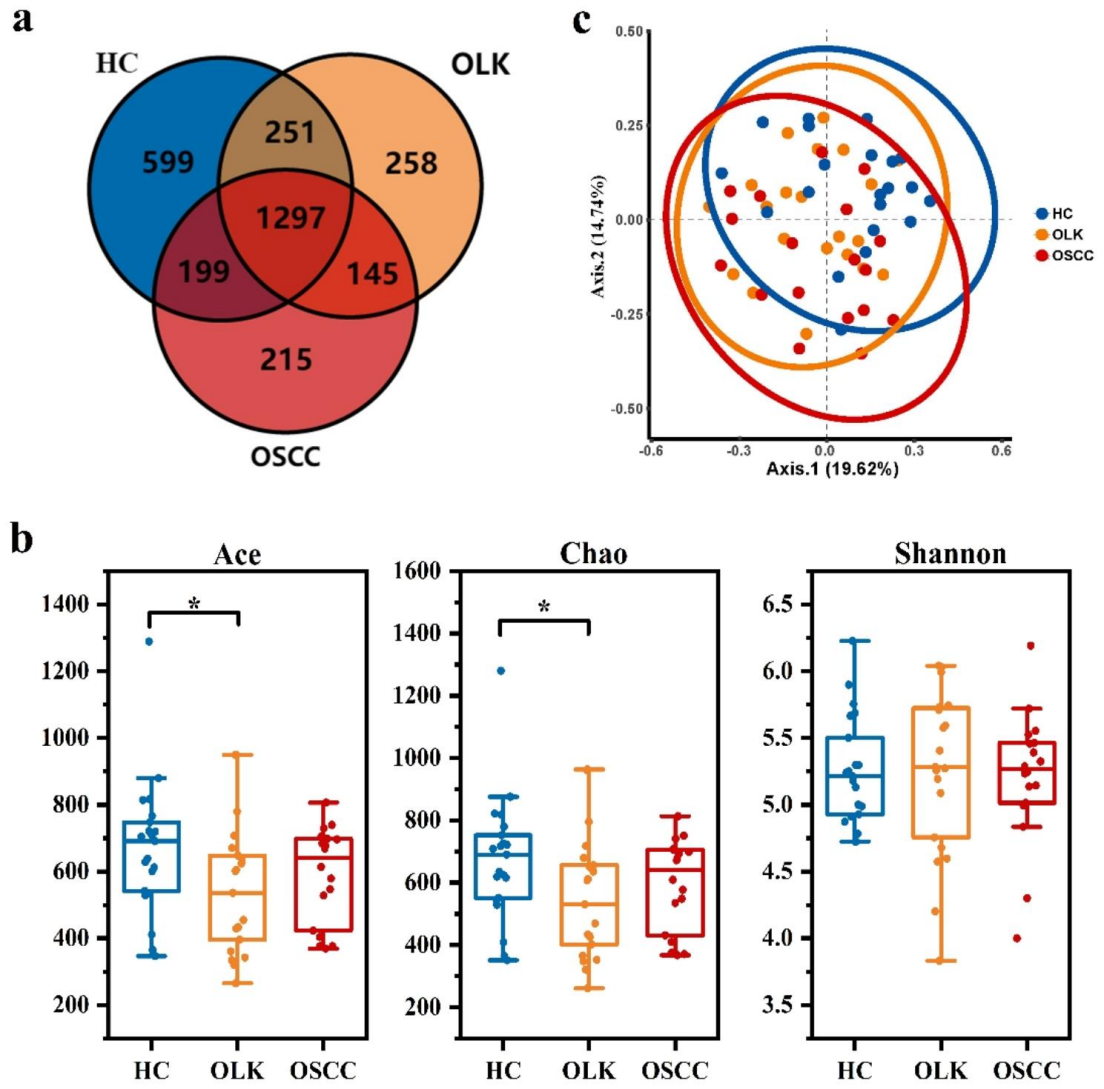
(**a**) Venn diagram of the three groups at the species level. (**b**) Ace, Chao and Shannon indices of the three groups. Wilcox tests were used to compare the significance of differences. The *p*-value between HC and OLK was 0.048 according to Chao. The *p*-value between HC and OLK was 0.043 according to Ace. *p < 0.05; **p < 0.01; ***p < 0.001. (**c**) Principal coordinate analysis (PCoA) of the three groups (PERMANOVA, F = 2.829, R^2^ = 0.09, *p*-value = 0.001). Axis 1 and Axis 2 explain 34.36% of the total variability among the three groups. Blue represents the healthy control group, red represents the OSCC group and orange represents the OLK group

First, metagenomics sequencing was used to investigate the composition of the oral microbiomes of participants. We drew a Venn diagram to summarise the common species among the three groups (Fig. 1a). We identified 2346 species in HC, 1951 in OLK and 1856 in OSCC. Interestingly only 258 species were presented in OLK and 215 species in OSCC, as compared to HC where a total of 599 species were presented.

Alpha-diversity in each group was evaluated using Shannon, Chao1 and Ace indices according to Wilcox tests. Significant differences were observed between OLK and HC groups according to Chao1 (*p* = 0.048) and Ace (*p* = 0.043), but no differences were found for Shannon (Fig. 1b). Beta-diversity was calculated by Bray-Curtis distance, and PCoA was performed to probe differences in microbiomes among groups. The results revealed significantly different distributions of salivary bacteria among the three groups (*p* = 0.001, PERMANOVA, Fig. 1c). What’s more, there were no differences between subjects with smoking or not, and between those with alcohol habit or not in three groups (HC with or without smoking habit: *p* = 0.347, PERMANOVA; OLK with or without smoking habit: *p* = 0.669, PERMANOVA; OSCC with or without smoking habit: *p* = 0.550, PERMANOVA; HC with or without alcohol habit: *p* = 0.801, PERMANOVA; OLK with or without alcohol habit: *p* = 0.752, PERMANOVA; OSCC with or without alcohol habit: *p* = 0.869, PERMANOVA).

#### Abundance comparison

**Fig. 2:**
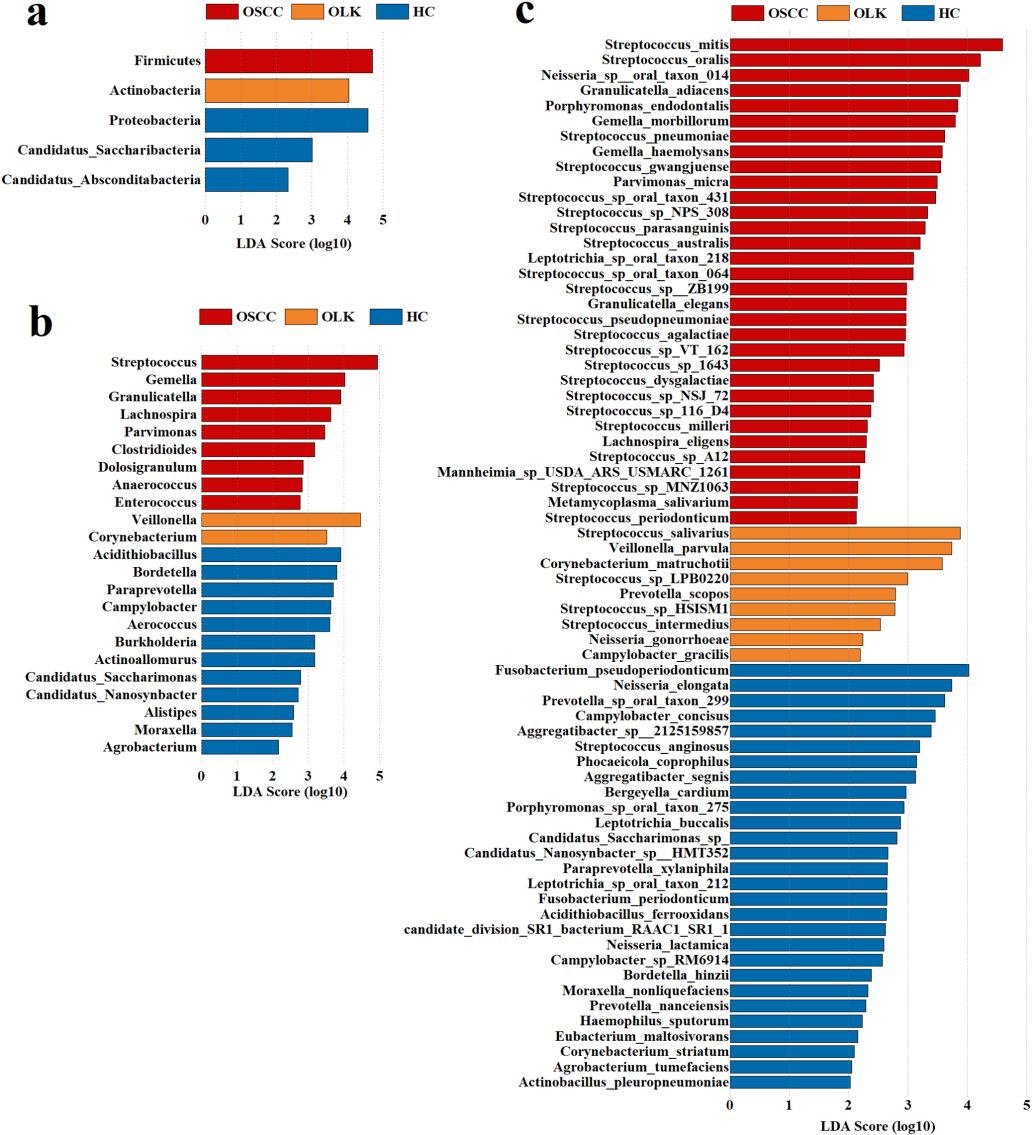
(**a**) Bacteria with significant differences in the three groups at the phylum level. (**b**) Bacteria with significant differences in the three groups at the genus level. (**c**) Bacteria with significant differences in the three groups at the species level. Blue represents the healthy control group, red represents the OSCC group and orange represents the OLK group. LDA Score > 2 was considered significant

Differentially abundant signatures of the three groups were assessed by LDA coupled with the LEfSe algorithm, and bacteria with an LDA score > 2 were considered significant. At the phylum level, Firmicutes were enriched in OSCC, while Actinobacteria were enriched in OLK (Fig. 2a). At the genus level (Fig. 2b), *Lachnospira*, *Granulicatella*, *Anaerococcus*, *Dolosigranulum*, *Gemella*, *Clostridioides*, *Streptococcus*, *Parvimonas* and *Enterococcus* were significantly enriched in OSCC, while *Veillonella* and *Corynebacterium* were enriched in OLK. A large proportion of the most abundant species belonged to the *Gemella* and *Streptococcus* genera (Fig. 2c).

#### Differences in bacterial composition associated with epithelial proliferation and dysplasia

**Fig. 3:**
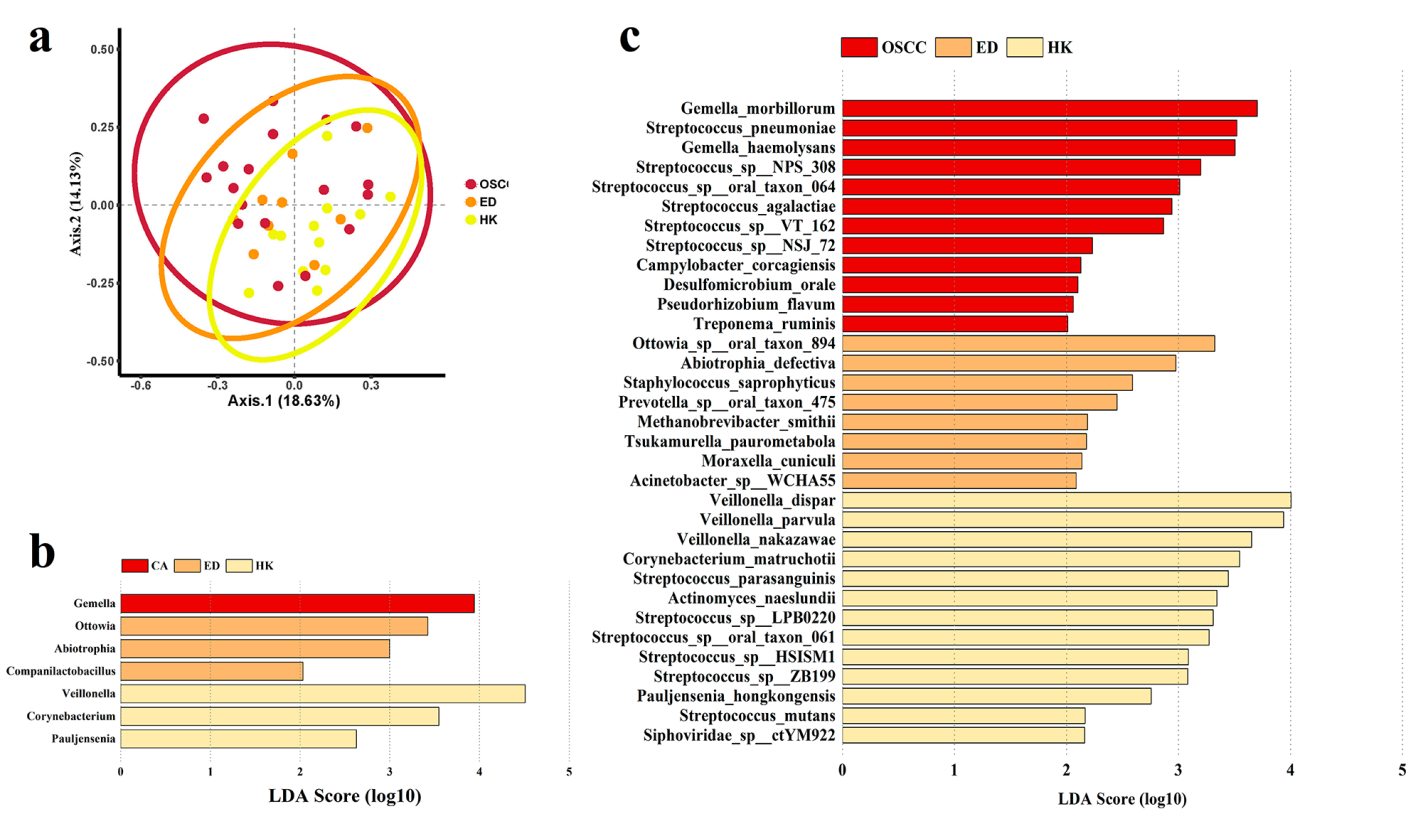
(**a**) Principal coordinate analysis (PCoA) among the three groups at the species level (PERMANOVA, F = 1.675, R^2^ = 0.112, *p* = 0.013). (**b**) Bacteria with differences among groups at the genus level. (**c**) Bacteria with differences among groups at the species level. Yellow represents the hyperplasia/keratosis (HK) group, red represents the OSCC group and orange represents the epithelial dysplasia (ED) group. LDA Score > 2 was considered significant

OLK subtypes were divided into hyperplasia/keratosis (HK) and epithelial dysplasia (ED), based on the degree of epithelial proliferation and dysplasia. PCoA was used to compare the diversity among HK, ED and OSCC (Fig. 3a), revealing significant differences at the species level (*p* = 0.013). No phylum showed significant differences according to LEfSe. At the genus level (Fig. 3b), *Gemella* was significantly enriched in the OSCC group. At the specie level (Fig. 3c), *Gemella morbillorum*, *Streptococcus pneumoniae*, *Gemella haemolysans*, *Streptococcus* sp. NPS 308, *Streptococcus* sp. oral taxon 064, *Streptococcus agalactiae*, *Streptococcus* sp. VT 162, *Streptococcus* sp. NSJ 72, *Campylobacter corcagiensis*, *Desulfomicrobium orale*, *Pseudorhizobium flavum* and *Treponema ruminis* were most abundant in OSCC.

#### Bacteria related to the progression in the development from epithelial proliferation to cancer

By comparing the degrees of epithelial proliferation and dysplasia in three groups, bacteria potentially related to the progression in the development of oral cancer were identified (Table 1a, 1b). The selecting criteria were: (1) LDA score > 2 for all three groups (HC, OLK and OSCC) and pathological grouping (HK, ED and OSCC); (2) the relative abundance increased progressively both in disease severity and epithelial dysplasia. *Gemella* matched the criteria at the genus level. At the species level, *Streptococcus* sp. NPS 308, *S. agalactiae*, *G. haemolysans* and *G. morbillorum* met the criteria. Although *G. morbillorum* was more abundant in HC than OLK, no significant difference was identified by the Dunn test. What’s more, all the bacteria selected showed no difference in patients with or without diabetes mellitus.

**Table 1a.** Selected genera potentially related to the progression in the development of oral cancer

Grouping	Sub-grouping	Genus	*p*-value
		*Gemella*	
Degree of epithelial dysplasia	HK	0.94%	0.001
	ED	0.83%	
	OSCC	2.59%	
Groups	HC	0.59%	< 0.001
	OLK	0.89%	
	OSCC	2.59%	

HK, hyperplasia/keratosis; ED, epithelial dysplasia; HC, healthy controls; OLK, oral leukoplasia; OSCC, oral squamous cell carcinoma

**Table 1b.** Selected species potentially related to the progression in the development of oral cancer

Species	Degrees of epithelial dysplasia			*p*-value	Groups			*p*-value
	HK	ED	OSCC		HC	OLK	OSCC	
*S.* sp. NPS 30*8*	0.04%	0.22%	0.43%	0.024	0.09%	0.12%	0.43%	0.029
*S. agalactiae*	0.06%	0.12%	0.23%	0.009	0.06%	0.08%	0.23%	0.001
*G. morbillorum*	0.06%	0.22%	1.20%	0.002	0.16%	0.13%	1.20%	0.001
*G. haemolysans*	0.27%	0.41%	0.91%	0.007	0.20%	0.33%	0.91%	< 0.001

HK, hyperplasia/keratosis; ED, epithelial dysplasia; HC, healthy controls; OLK, oral leukoplasia; OSCC, oral squamous cell carcinoma; *S.* sp. NPS 30*8*, *Streptococcus* sp. NPS 308; *S. agalactiae*, *Streptococcus agalactiae*; *G. morbillorum*, *Gemella morbillorum*; *G. haemolysans*, *Gemella haemolysans*

### Functional analysis

#### Functional comparison

**Fig. 4:**
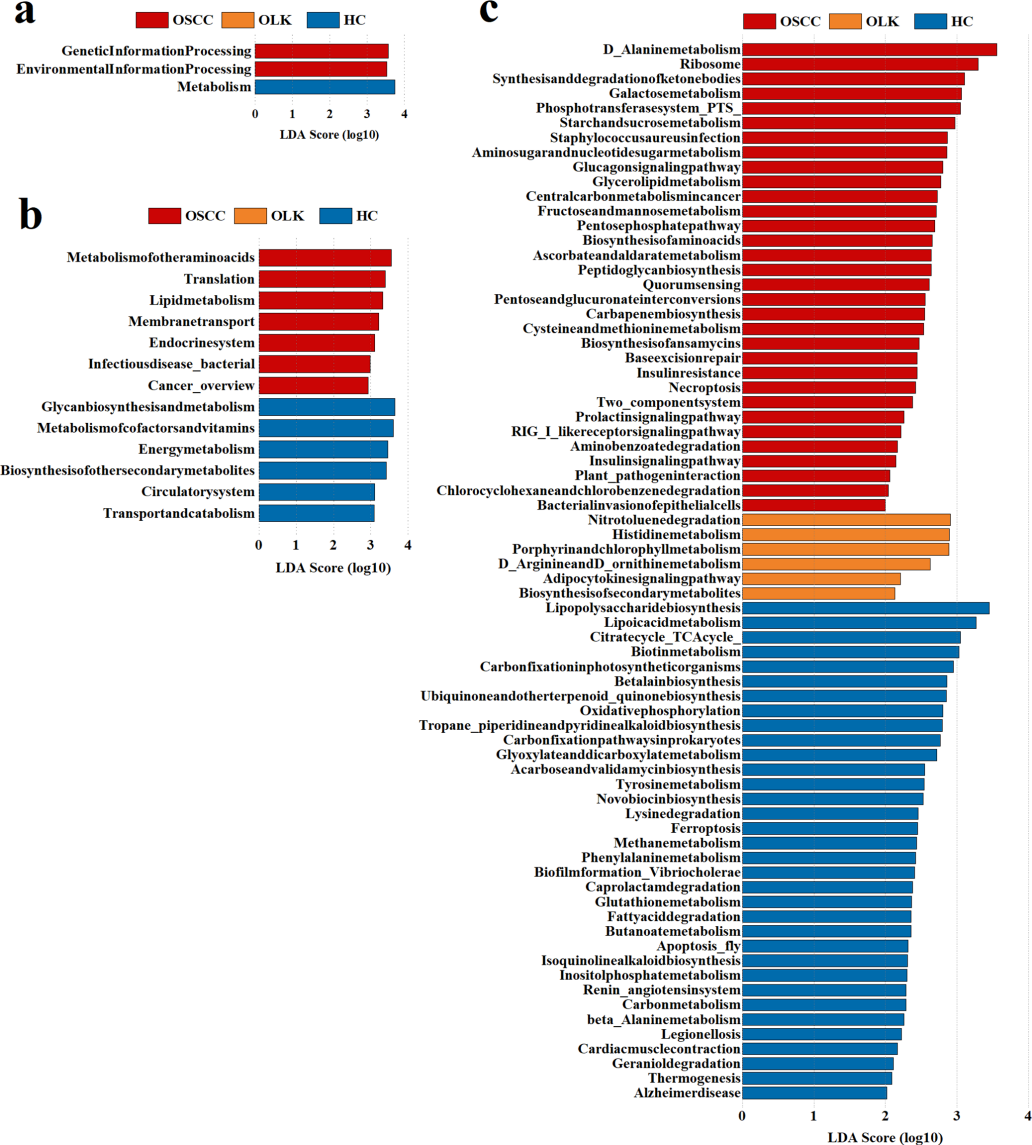
(**a**) Functional differences of bacteria among groups at KEGG level (1) (**b**) Functional differences of bacteria among groups at KEGG level (2) (**c**) Functional differences of bacteria among groups at KEGG level (3) Blue represents the healthy control group, red represents the OSCC group and orange represents the OLK group. LDA Score > 2 was considered significant

Functional changes in the oral microbiomes across the three groups were explored using the KEGG database. The results were analysed by LEfSe (Fig. 4a, b and c) and Dunn tests to reveal differences among groups. At level 1, genetic information processing and environmental information processing were significantly increased in OSCC, while metabolism was significantly increased in HC. At level 2, metabolism of other amino acids (Metabolism), translation (Genetic Information Processing), lipid metabolism (Metabolism), membrane transport (Environmental Information Processing), endocrine system (Organismal Systems), infectious disease bacterial (Human Diseases) and cancer overview (Human Diseases) were enriched in OSCC. Meanwhile in HC, transport and catabolism (Cellular Processes), circulatory system (Organismal Systems), biosynthesis of other secondary metabolites (Metabolism), energy metabolism (Metabolism), metabolism of cofactors and vitamins (Metabolism), glycan biosynthesis and metabolism (Metabolism) were significantly enriched.

### Possible risk functions of bacteria related to oral cancer

**Fig. 5:**
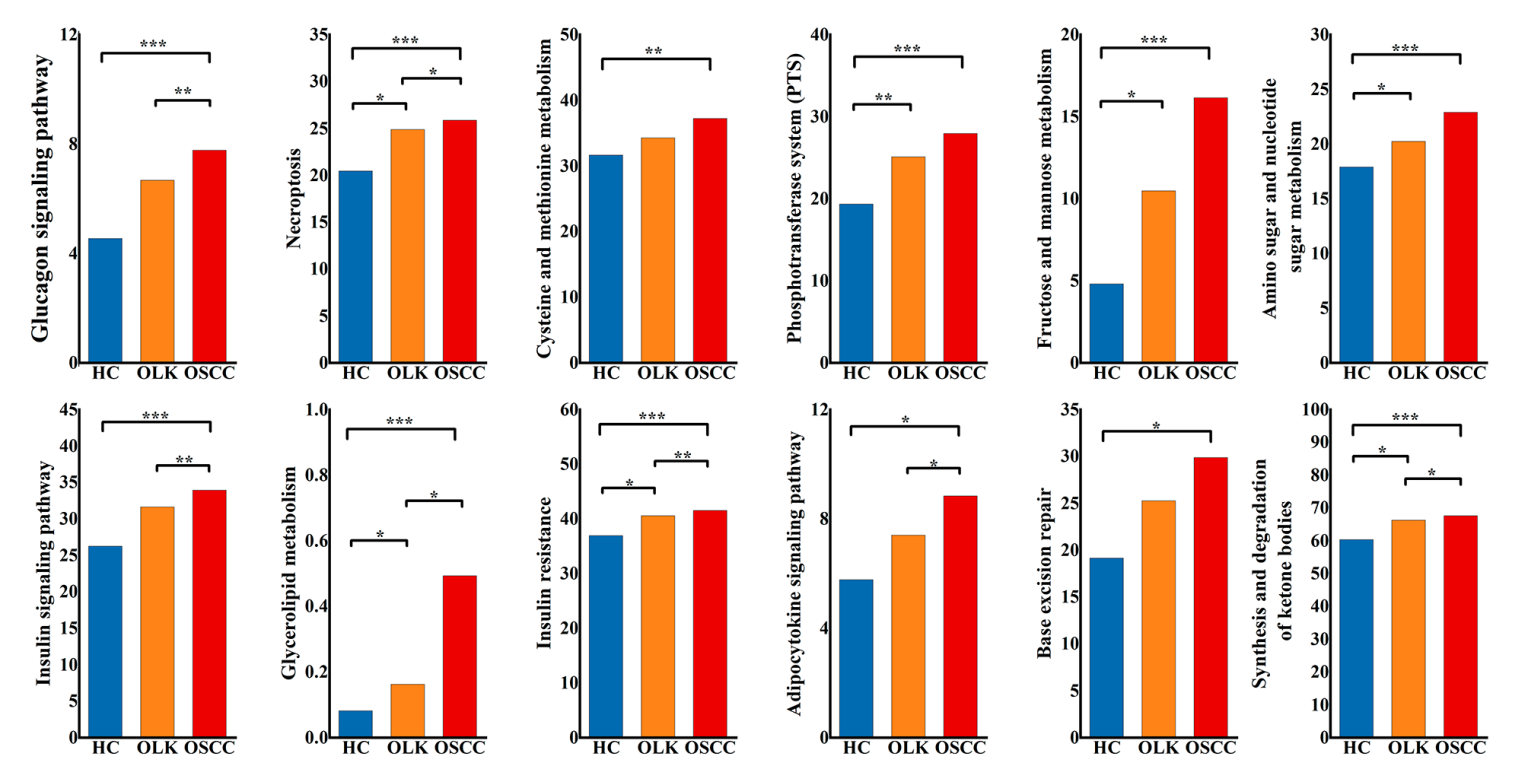
Differences in metabolic functions of bacteria potentially related to OSCC. *p < 0.05; **p < 0.01; ***p < 0.001. All the above *p* values in this figure were adjusted. Blue represents the healthy control group, red represents the OSCC group and orange represents the OLK group

Functions (Fig. 5) were selected according to the following conditions: (1) LDA > 2 in the three groups (HC, OLK and OSCC); (2) the relative abundance increased progressively with disease severity; (3) the abundance of functions was significantly higher in OSCC. Significantly different pathways were found related to lipid metabolism (Metabolism) and carbohydrate metabolism (Metabolism), including glycerolipid metabolism (level 2: Lipid metabolism; level 1: Metabolism), galactose metabolism (level 2: Carbohydrate metabolism; level 1: Metabolism), fructose and mannose metabolism (level 2: Carbohydrate metabolism; level 1: Metabolism), cysteine and methionine metabolism (level 2: Amino acid metabolism; level 1: Metabolism) and insulin signaling pathway (level 2: Endocrine system; level 1: Organismal Systems).

### Possible associations between selected functions and bacteria

**Fig. 6:**
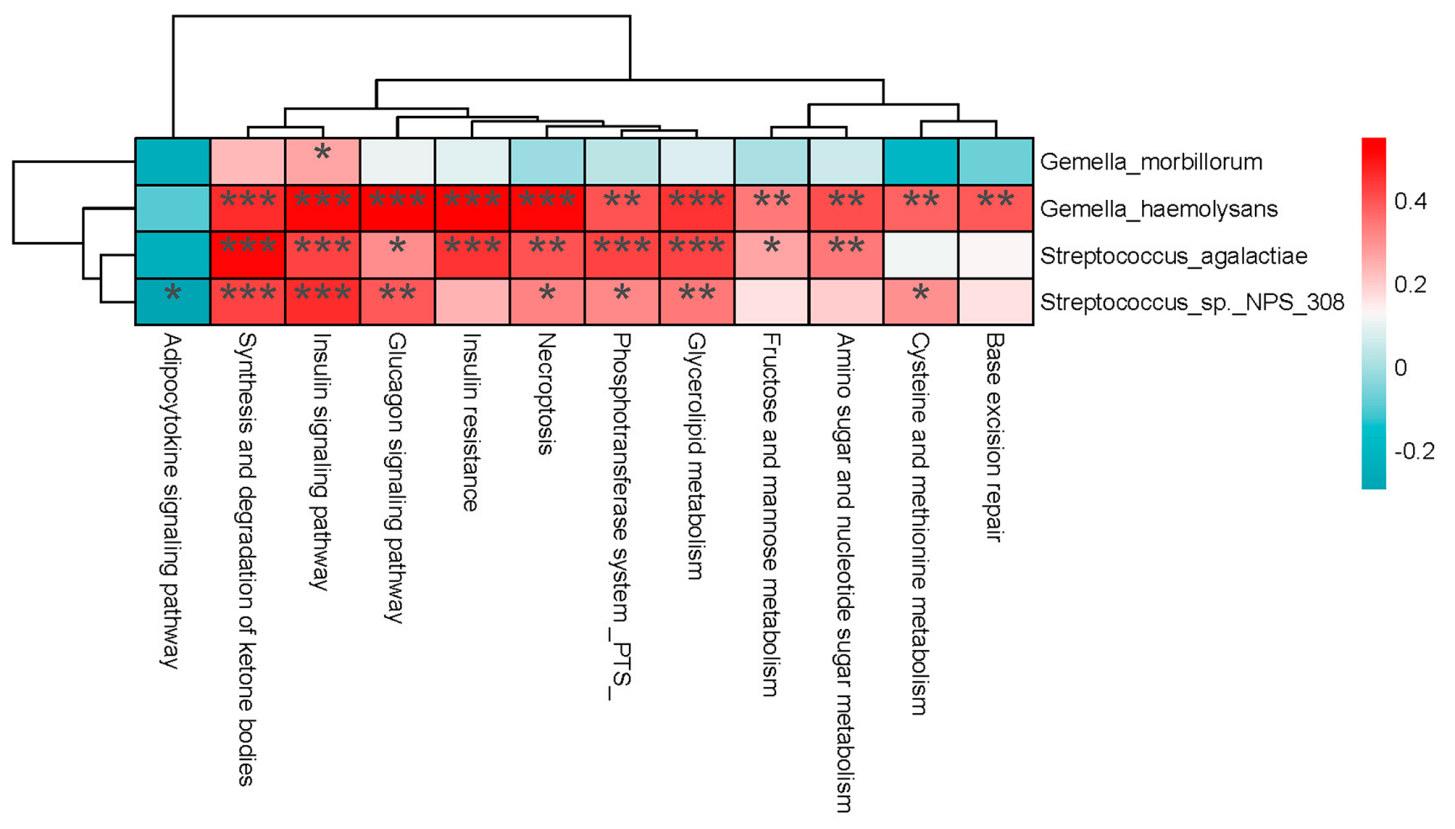
Heatmap showing correlations between microbiomes and metabolic functions that may be associated with OSCC

In order to identify possible associations between selected functions and microbiomes, Spearman correlation analysis was performed to generate a heatmap (Fig. 6). The results showed that *G. haemolysans* was significantly correlated with the above 12 selected metabolic pathways.

## Discussion

This study focused on the relationship between microbiomes and oral carcinogenesis. OSCC and OLK patients were included to represent a transition from normal oral mucosa to oral cancer. The oral microbiome is one of the most complex microbial communities in the human body, and numerous studies have reported an association between ecological dysbiosis and oral cancer development [33]. However, the results of different studies vary considerably. Li et al. explored associations among controls, OSCC groups and precancerous lesions with gargle samples using metagenomic analysis [34]. They found that Bacteroidetes at the phylum level and *Prevotella* and *Peptostreptococcus* at the genus level were significantly enriched in OSCC compared with precancerous lesions and HC, in contrast with the results of the present work. However, the authors did not clarify subtypes of precancerous lesions. In our study, the genus *Gemella* was found to be enriched in OSCC samples, consistent with previous reports that *Gemella* was more abundant in tumour tissue samples than non-tumour tissue samples from the same OSCC patients [35]. Moreover, *Gemella* was found to be significantly more abundant in active oral malignant lesions (before surgical resection defined as active lesions) than healthy controls and those without active lesions (undergone surgical resection defined as without active lesions) based on 16S rDNA analysis of saliva samples [36]. Additionally, 16S rRNA analysis of biopsy samples of patients with colorectal cancer (CRC), patients with adenomatous polyps, and healthy controls in Norway indicated that *Gemella* was enriched in cancer patients compared with the other two groups [37]. Similar to our current findings, these studies suggested that *Gemella* may be indicative of metabolic processes occurring during carcinogenesis. At the species level, *Streptococcus* sp. NPS 308, *S. agalactiae* and *G. haemolysans* were identified that may be associated with the process of oral carcinogenesis. Although *G. morbillorum* was more abundant in the HC group than the OLK group, Dunn test results indicated no difference between these two groups, hence this species may be associated with carcinogenesis. Many studies found that *G. morbillorum* and *G. haemolysans* were strongly associated with tumour sites, according to 16S rDNA analysis of biopsy samples [35, 38, 39]. Moreover, the abundance of these bacteria increased with the invasion depth of OSCC in biopsy samples [40]. Another study found that C-C motif chemokine 2 (CCL2) levels correlated with CRC presence and with the abundance of *G. haemolysans* in the colon of CRC patients, according to linear multiple regression analysis [41], which indicated that *G. haemolysans* may be associated to immune response in oncogenesis. Although an association between *S. agalactiae* and cancer has not been reported, it has been linked to inflammation in the form of sepsis and meningitis in neonates [42]. While it was found that *Streptococci* were saccharolytic producing short chain organic acid from carbohydrates, which may contribute to the acidic microenvironment of cancers [35, 43].

Metagenomic sequencing provided additional information. When analysing KEGG pathways, the main downregulated pathways were related to metabolism, while genetic information processing and environmental information processing were significantly upregulated in the OSCC group. Furthermore, unusual metabolic including lipid metabolism (Metabolism), carbohydrate metabolism (Metabolism), glycan biosynthesis and metabolism (Metabolism), synthesis and degradation of ketone bodies (level 2: Lipid metabolism; level 1: Metabolism), cysteine and methionine metabolism (level 2: Amino acid metabolism; level 1: Metabolism) were identified. Upregulation of the phosphotransferase system (PTS) (level 2: Membrane transport; level 1: Environmental Information Processing) and fructose and mannose metabolism (level 2: Carbohydrate metabolism; level 1: Metabolism) might reflect a decrease in sugar sources on the tumour surface, since increased glucose absorbance is important for OSCC cell survival [44]. Metabolism of proteins may be associated with CRC, and some subtypes of cysteine and methionine (level 2: Amino acid metabolism; level 1: Metabolism) are increased in CRC patients [45], consistent with our studies. Microbial sulphidogenesis produces genotoxic hydrogen sulphide (H2S) in the human colon using inorganic sulphate and amino acids such as cysteine and methionine, which may contribute to the pathogenesis of CRC [46, 47]. Synthesis and degradation of sulfur-containing amino acids cysteine and methionine metabolism were found significantly higher in OSCC group from OLK and HC groups in our study. They were reported susceptible to reactive oxygen species (ROS) and reactive chlorine species (RCS), which can damage proteins [48]. In addition, the metabolism of cysteine and methionine produces intermediate metabolites that form the cellular antioxidant systems, control both intercellular and intracellular signals, and enable the epigenetic control of gene expressions, all of which play a role in oncogenesis [49].

Our study had some limitations. First, the number of samples was relatively low, and more cases should be investigated to confirm the results. Secondly, all patients were Chinese, hence there may be population bias. Besides, our study analyzed the saliva microbiota, which may not reflect the exact conditions on the surface or in the oral lesions. And the lack of adjusting for multiple testing may increase the probability of false positive in LEfSe. Future studies are needed to address these limitations, such as multi-centre studies with a large number of patients and multi-racial and multi-ethnic diverse populations. And more studies with samples directly obtained from oral lesions such as cotton swabs and tissues are needed in the future. Additionally, in order to explore associations between metabolism and diseases, further studies should be carried out on more diabetes mellitus patients and dyslipidemia patients with OSCC or OLK, for which biochemical indices and treatments are required to glean basic information.

## Conclusion

We observed significant salivary bacterial compositional and functional changes among OSCC, OLK and HC groups at different taxonomic levels. In addition, we found that the microbiome composition differed with varying degrees of epithelial dysplasia. Shifts in the oral microbiota involved specific metabolic pathways linked to OLK or OSCC. Further studies are needed to confirm the associations between changes in the oral microbiome, and to provide new insight into the pathogenesis of OLK and OSCC.

### Electronic supplementary material

Below is the link to the electronic supplementary material.

**Additional file 1**: Supplementary Table 1.

## Data Availability

The datasets used and/or analyzed during the current study are available from the corresponding author on reasonable request.

## Abbreviations

OSCC: Oral squamous cell carcinoma
OLK: Oral leukoplakia
HC: Healthy controls
KEGG: Kyoto Encyclopedia of Genes and Genomes
G. haemolysans: Gemella haemolysans
OPMDs: Oral potentially malignant disorders
MT: Malignant transformation
16 S rRNA: 16 S ribosomal RNA
CTAB: Cetyltrimethylammonium bromide
HK: Hyperplasia/keratosis
ED: Epithelial dysplasia
CCL2: C-C motif chemokine 2
CRC: Colorectal cancer
PTS: Phosphotransferase system
H2S: Hydrogen sulphide

## References

[1] Sung H, Ferlay J, Siegel RL, Laversanne M, Soerjomataram I, Jemal A (2021). Global Cancer statistics 2020: GLOBOCAN estimates of incidence and Mortality Worldwide for 36 cancers in 185 countries. Cancer J Clin.

[2] Warnakulasuriya S (2009). Global epidemiology of oral and oropharyngeal cancer. Oral Oncol.

[3] Al-Hebshi NN, Borgnakke WS, Johnson NW (2019). The microbiome of oral squamous cell carcinomas: a functional perspective. Curr Oral Health Rep.

[4] Aguirre-Urizar JM, Lafuente‐Ibáñez de Mendoza I, Warnakulasuriya S (2021). Malignant transformation of oral leukoplakia: systematic review and meta‐analysis of the last 5 years. Oral Dis.

[5] Dos Santos ES, Pérez-de‐Oliveira ME, Normando AGC, Gueiros LAM, Rogatto SR, Vargas PA (2022). Systemic conditions associated with increased risk to develop oral squamous cell carcinoma: systematic review and meta‐analysis. Head Neck.

[6] Smoot DT (1997). How does Helicobacter pylori cause mucosal damage? Direct mechanisms. Gastroenterology.

[7] Liu H, Liang H, Li D, Wang M, Li Y (2022). Association of Cervical Dysbacteriosis, HPV Oncogene expression, and cervical lesion progression. Microbiol Spectr.

[8] Wang X, Jia Y, Wen L, Mu W, Wu X, Liu T (2021). Porphyromonas gingivalis promotes colorectal carcinoma by activating the hematopoietic NLRP3 Inflammasome. Cancer Res.

[9] Kostic AD, Gevers D, Pedamallu CS, Michaud M, Duke F, Earl AM (2012). Genomic analysis identifies association of Fusobacterium with colorectal carcinoma. Genome Res.

[10] Winkler ES, Thackray LB (2019). A long-distance relationship: the commensal gut microbiota and systemic viruses. Curr Opin Virol.

[11] Perera M, Al-hebshi NN, Perera I, Ipe D, Ulett GC, Speicher DJ (2018). Inflammatory bacteriome and oral squamous cell carcinoma. J Dent Res.

[12] Tornesello ML, Zammit AP, Sinha R, Cooper CL, Perry CFL, Frazer IH et al. Examining the contribution of Smoking and HPV towards the etiology of oral cavity squamous cell carcinoma using high-throughput sequencing: a prospective observational study. PLoS ONE. 2018;13(10). 10.1371/journal.pone.0205406 PMC6181346 30308005

[13] Bugshan A, Farooq I. Oral squamous cell carcinoma: Metastasis, potentially associated malignant disorders, etiology and recent advancements in diagnosis. F1000Research. 2020;9. 10.12688/f1000research.22941.1 PMC7194458 32399208

[14] Vadovics M, Ho J, Igaz N, Alfoldi R, Rakk D, Veres E (2022). Candida albicans enhances the progression of oral squamous cell Carcinoma in Vitro and in vivo. mBio.

[15] Li R, Xiao L, Gong T, Liu J, Li Y, Zhou X et al. Role of oral microbiome in oral oncogenesis, Tumor progression and Metastasis. Mol Oral Microbiol. 2022. 10.1111/omi.12403 36420924

[16] Hu X, Zhang Q, Hua H, Chen F (2016). Changes in the salivary microbiota of oral leukoplakia and Oral cancer. Oral Oncol.

[17] Gopinath D, Kunnath Menon R, Chun Wie C, Banerjee M, Panda S, Mandal D et al. Salivary bacterial shifts in oral leukoplakia resemble the dysbiotic Oral cancer bacteriome. J Oral Microbiol. 2020;13(1). 10.1080/20002297.2020.1857998 PMC7734041 33391629

[18] Schmidt BL, Kuczynski J, Bhattacharya A, Huey B, Corby PM, Queiroz EL (2014). Changes in abundance of oral microbiota associated with Oral cancer. PLoS ONE.

[19] Chattopadhyay I, Verma M, Panda M. Role of oral Microbiome signatures in diagnosis and prognosis of Oral Cancer. Technol Cancer Res Treat. 2019;18. 10.1177/1533033819867354 PMC6676258 31370775

[20] Solbiati J, Frias-Lopez J (2018). Metatranscriptome of the oral Microbiome in Health and Disease. J Dent Res.

[21] Krishnan K, Chen T, Paster BJ (2017). A practical guide to the oral microbiome and its relation to health and Disease. Oral Dis.

[22] Speight PM, Khurram SA, Kujan O (2018). Oral potentially malignant disorders: risk of progression to malignancy. Oral Surg Oral Med Oral Pathol Oral Radiol.

[23] Ranganathan K, Kavitha L (2019). Oral epithelial dysplasia: classifications and clinical relevance in risk assessment of oral potentially malignant disorders. J Oral Maxillofac Pathol.

[24] Navazesh M (1993). Methods for collecting saliva. Ann N Y Acad Sci.

[25] Bolger AM, Lohse M, Usadel B (2014). Trimmomatic: a flexible trimmer for Illumina sequence data. Bioinformatics.

[26] Langmead B, Salzberg SL (2012). Fast gapped-read alignment with Bowtie 2. Nat Methods.

[27] Wood DE, Salzberg SL (2014). Kraken: ultrafast metagenomic sequence classification using exact alignments. Genome Biol.

[28] Lu J, Breitwieser FP, Thielen P, Salzberg SL (2017). Bracken: estimating species abundance in metagenomics data. Peerj Comput Sci.

[29] Cotillard A, Kennedy SP, Kong LC, Prifti E, Pons N, Le Chatelier E (2013). Erratum: Corrigendum: dietary intervention impact on gut microbial gene richness. Nature.

[30] Buttigieg PL, Ramette A (2014). A guide to statistical analysis in microbial ecology: a community-focused, living review of multivariate data analyses. FEMS Microbiol Ecol.

[31] Franzosa EA, McIver LJ, Rahnavard G, Thompson LR, Schirmer M, Weingart G (2018). Species-level functional profiling of metagenomes and metatranscriptomes. Nat Methods.

[32] von Elm E, Altman DG, Egger M, Pocock SJ, Gøtzsche PC, Vandenbroucke JP (2014). The strengthening the reporting of Observational studies in Epidemiology (STROBE) Statement: guidelines for reporting observational studies. Int J Surg.

[33] Schwabe RF, Jobin C (2013). The microbiome and cancer. Nat Rev Cancer.

[34] Li Z, Chen G, Wang P, Sun M, Zhao J, Li A et al. Alterations of the oral microbiota profiles in Chinese patient with Oral Cancer. Front Cell Infect Microbiol. 2021;11. 10.3389/fcimb.2021.780067 PMC8696029 34956932

[35] Pushalkar S, Ji X, Li Y, Estilo C, Yegnanarayana R, Singh B (2012). Comparison of oral microbiota in Tumor and non-tumor tissues of patients with oral squamous cell carcinoma. BMC Microbiol.

[36] Granato DC, Neves LX, Trino LD, Carnielli CM, Lopes AFB, Yokoo S et al. Meta-omics analysis indicates the saliva microbiome and its proteins associated with the prognosis of Oral cancer patients. Biochimica et Biophysica Acta (BBA) - proteins and proteomics. 2021;1869(8). 10.1016/j.bbapap.2021.140659 33839314

[37] Senthakumaran T, Moen AEF, Tannaes TM, Endres A, Brackmann SA, Rounge TB (2023). Microbial dynamics with CRC progression: a study of the mucosal microbiota at multiple sites in cancers, adenomatous polyps, and healthy controls. Eur J Clin Microbiol Infect Dis.

[38] Zhang L, Liu Y, Zheng HJ, Zhang CP. The oral Microbiota May have influence on Oral Cancer. Front Cell Infect Microbiol. 2020;9. 10.3389/fcimb.2019.00476 PMC6974454 32010645

[39] Stasiewicz M, Karpiński TM (2022). The oral microbiota and its role in carcinogenesis. Sem Cancer Biol.

[40] Liu Y, Li Z, Qi Y, Wen X, Zhang L. Metagenomic analysis reveals a changing Microbiome Associated with the depth of Invasion of oral squamous cell carcinoma. Front Microbiol. 2022;13. 10.3389/fmicb.2022.795777 PMC8863607 35222330

[41] Nardelli C, Granata I, Nunziato M, Setaro M, Carbone F, Zulli C (2021). 16S rRNA of mucosal Colon microbiome and CCL2 circulating levels are potential biomarkers in Colorectal Cancer. Int J Mol Sci.

[42] Walker EA, Port GC, Caparon MG, Janowiak BE. Glutathione synthesis contributes to virulence of Streptococcus agalactiae in a murine model of Sepsis. J Bacteriol. 2019;201(20). 10.1128/JB.00367-19 PMC6755738 31331978

[43] Lunt SJ, Chaudary N, Hill RP (2009). The Tumor microenvironment and metastatic Disease. Clin Exp Metastasis.

[44] Eckert AW, Wickenhauser C, Salins PC, Kappler M, Bukur J, Seliger B. Correction to: clinical relevance of the Tumor microenvironment and immune Escape of oral squamous cell carcinoma. J Translational Med. 2018;16(1). 10.1186/s12967-018-1407-9 PMC5830086 29486780

[45] Coker OO, Liu C, Wu WKK, Wong SH, Jia W, Sung JJY et al. Altered gut metabolites and microbiota interactions are implicated in colorectal carcinogenesis and can be non-invasive diagnostic biomarkers. Microbiome. 2022;10(1). 10.1186/s40168-021-01208-5 PMC8862353 35189961

[46] Wolf PG, Cowley ES, Breister A, Matatov S, Lucio L, Polak P (2022). Diversity and distribution of sulfur metabolic genes in the human gut microbiome and their association with Colorectal cancer. Microbiome.

[47] Blachier F, Beaumont M, Kim E (2019). Cysteine-derived hydrogen sulfide and gut health: a matter of endogenous or bacterial origin. Curr Opin Clin Nutr Metab Care.

[48] Ezraty B, Gennaris A, Barras F, Collet JF (2017). Oxidative stress, protein damage and repair in bacteria. Nat Rev Microbiol.

[49] Ward NP, DeNicola GM (2019). Sulfur metabolism and its contribution to malignancy. Int Rev Cell Mol Biol.

