# Supplementary material for: Compositional and functional changes in the salivary microbiota related to oral leukoplakia and oral squamous cell carcinoma: a case control study
Source: BMC Oral Health. 2023 Dec 19;23:1021. doi: 10.1186/s12903-023-03760-y (PMC10731685; doi:10.1186/s12903-023-03760-y)
Supplement: Supplementary file 1 — Additional file 1: Supplementary Table 1. [file 12903_2023_3760_MOESM1_ESM.docx]

**Supplementary materials**

**Supplementary Table 1: Basic characteristics of the study subjects**

| **Parameter** | **HC** | **OLK** | **OSCC** | ***p*-value** |
| --- | --- | --- | --- | --- |
| **Total number of subjects** | 21 | 21 | 18 | - |
| **Average age (years)** | 48.81 ± 12.38 | 59.24 ± 12.30 | 54.11 ± 15.43 | 0.048 |
| **Sex (male)** | 7 | 4 | 8 | 0.237 |
| **Sex (female)** | 14 | 17 | 10 | - |
| **Average PD (mm)** | 3.52 ± 0.52 | 3.94 ± 0.86 | 4.14 ± 0.75 | 0.053 |
| **BI** | 2.67 ± 0.86 | 3.29 ± 0.90 | 3.50 ± 0.71 | 0.007 |
| **CI** | 2.14 ± 0.73 | 1.95 ± 0.87 | 2.50 ± 0.62 | 0.096 |
| **DM (Yes)** | 0 | 6 | 3 | - |
| **DM (No)** | 21 | 15 | 15 | 0.036 |

HC, healthy controls; OLK, oral leukoplakia; OSCC, oral squamous cell carcinoma; PD, probing depth; BI, bleeding index; CI, calculus index; DM, diabetes mellitus. Results are presented as mean ± standard deviation. One-way ANOVA was used to compare the significance of differences in age among the three groups. Kruskal-Wallis tests were conducted to compare differences in PD, BI and CI (p <0.05 was considered significant).
